# Development of a Deep Learning–Based Feedback Model to Assist Medical Students Learning Renal Ultrasound Acquisition: Mixed Methods Study

**DOI:** 10.2196/72110

**Published:** 2026-03-09

**Authors:** Andy Cheuk Nam Hwang, Rahul Singh, Elizabeth Ann Barrett, Peng Cao, Varut Vardhanabhuti, Pauline Yeung Ng, Gordon Tin Chun Wong, Michael Tiong Hong Co, Elaine Yuen-Phin Lee

**Affiliations:** 1 Department of Diagnostic Radiology, School of Clinical Medicine, Li Ka Shing Faculty of Medicine, The University of Hong Kong, 21 Sassoon Rd, Pok Fu Lam, Hong Kong, China, +852 22553307; 2 Academic Unit of Human Communication, Learning, and Development, Faculty of Education, The University of Hong Kong, Hong Kong, China; 3 Critical Care Medicine Unit, School of Clinical Medicine, Li Ka Shing Faculty of Medicine, The University of Hong Kong, Hong Kong, China; 4 Department of Anaesthesiology, School of Clinical Medicine, Li Ka Shing Faculty of Medicine, The University of Hong Kong, Hong Kong, China; 5 Department of Surgery, School of Clinical Medicine, Li Ka Shing Faculty of Medicine, The University of Hong Kong, Hong Kong, China

**Keywords:** point-of-care ultrasound, renal ultrasound learning, deep learning, convolutional neural networks, automated feedback system

## Abstract

**Background:**

Point-of-care ultrasound training is being increasingly integrated into undergraduate medical education, leading to a substantial demand for trained faculty to provide instruction and feedback.

**Objective:**

This study aimed to develop an adjunct tool, a deep learning–based feedback model, to facilitate student learning.

**Methods:**

Renal ultrasound images (N=2807) were used to train a cascaded deep learning–based feedback model that classified images into three categories: optimal, suboptimal, and incorrect. Suboptimal images were further subcategorized as images with artifact, incorrect gain, and/or incorrect positioning. The model was deployed among year 5 medical students receiving bedside ultrasound training, who were invited to upload renal ultrasound images to an online platform for automated image quality grading and feedback. A mixed methods analysis was used to evaluate students’ learning experience. Focus group interviews were organized to qualitatively analyze the successes and challenges of implementation. Quantitative analysis was based on responses to a 5-point Likert scale questionnaire and performance on the objective structured clinical examination (OSCE). Objective structured clinical examination scores were compared with mean OSCE scores from the 2 years preceding implementation of the deep learning–based feedback model.

**Results:**

Focus group interviews identified that the deep learning–based feedback model encouraged self-regulated learning but also recognized that discordant curricular design and hardware limitations impeded its use. The 11-item online questionnaire had a response rate of 42.4% (98/231 students). Among respondents, 32% (31/98) to 48% (47/98) found the model helpful in assisting ultrasound training (Likert score of 4‐5 for items 1-3), while 49% (48/98) to 76% (74/98) were satisfied with its usability and their interaction with the model (Likert score of 4‐5 for items 4-11). The mean OSCE score was 9.73 (SD 0.76) out of 10, compared with mean scores of 9.35 (SD 1.03; *P*=.06) and 9.45 (SD 0.97; *P*=.15) out of 10 in the 2 individual years preceding implementation of the model.

**Conclusions:**

A cascaded deep learning–based feedback model was developed and deployed among year 5 medical students receiving bedside ultrasound training, with positive learner responses and enhanced self-regulated learning. The innovation was associated with increased student engagement and improved ultrasound skill acquisition among novice learners.

## Introduction

Point-of-care ultrasound (POCUS) refers to the use of ultrasound imaging to facilitate clinical diagnosis and management while patients are being treated. Substantial evidence supports POCUS in aiding diagnosis and improving bedside procedures and clinical management [1-3]. To address this clinical need, the next generation of clinicians involved in acute care should master skills such as image acquisition, interpretation, and clinical integration of POCUS findings. Therefore, ultrasound training is increasingly being introduced and incorporated into undergraduate medical education (UME). In 2019, a survey conducted in the United States reported that 72.6% of 168 accredited medical schools included an ultrasound curriculum in their UME [4]. Cross-specialty POCUS training has been shown to augment physical examination skills among undergraduate learners and to lay the foundation for future postgraduate training [5,6]. However, the demand for trained faculty and tutors remains a major barrier to implementing ultrasound curricula in UME; 63% of medical schools in the United States reported that they did not have trained faculty for POCUS instruction [7].

Ultrasound imaging across different organs has variable learning curves, reflecting different rates of skill acquisition. Among these, renal ultrasound has a relatively longer learning curve [8], suggesting that it is moderately challenging to students. This underscores the need for additional practice and faculty guidance to support skill development. Different pedagogical approaches in renal and abdominal ultrasound training have demonstrated comparable improvements in learner proficiency [9,10]. This suggests that renal ultrasound skill acquisition is adaptable to different pedagogical approaches to support student learning.

Feedback is important to student learning because it facilitates self-reflection, understanding, and future improvement, particularly in skill acquisition and mastery [11]. Effective feedback positions students as active learners in the feedback process, empowering them to understand their performance and develop evaluative judgment to improve learning [12]. Feedback is most beneficial when immediate, external feedback on a specific task is provided [11]. Students benefit from multiple opportunities to engage with feedback from different sources [13,14]. However, feedback is often neither adequately provided nor delivered effectively [15]. This challenge is exacerbated in large-scale higher education [16,17]. A technology-enabled feedback process may streamline practice and address challenges associated with low instructor-to-student ratios and the inability to provide on-demand feedback [18]. It can customize students’ learning by allowing them to determine when and where their learning occurs and to control their pace. It may support both blended and adaptive learning strategies and provide a flexible learning environment not limited to workshops and bedside teaching sessions [19-22]. This approach may also promote self-regulated learning [23], an important conceptual framework in education. Self-reflection is an invaluable step in preparing students for the next phase of the learning cycle [24].

A deep learning model based on convolutional neural networks (CNNs) is a promising approach for image classification [25]. A number of pretrained CNNs with strong general performance, such as ResNet [26] and SENet [27], have been developed. These pretrained CNNs can be fine-tuned and trained for classification tasks involving medical images. In this study, we hypothesized that a deep learning–based feedback model could enhance learner motivation and support self-regulated learning in renal ultrasound acquisition. Accordingly, the aims of this study were to develop a deep learning–based feedback model and evaluate its acceptance and impact on renal ultrasound acquisition skills.

## Methods

### Ethical Considerations

This study was approved by the Institutional Review Board of the University of Hong Kong/Hospital Authority Hong Kong West Cluster (HKU/HA HKW IRB; UW 22‐797). This study was conducted in accordance with the Declaration of Helsinki and the International Council for Harmonisation Good Clinical Practice guidelines. Students were invited to complete the questionnaire voluntarily and anonymously. All data generated and analyzed in this study were anonymized. The requirement for informed consent was waived by the HKU/HA HKW Institutional Review Board. No compensation was provided.

### Development of a Deep Learning Feedback Model

**Figure 1.**
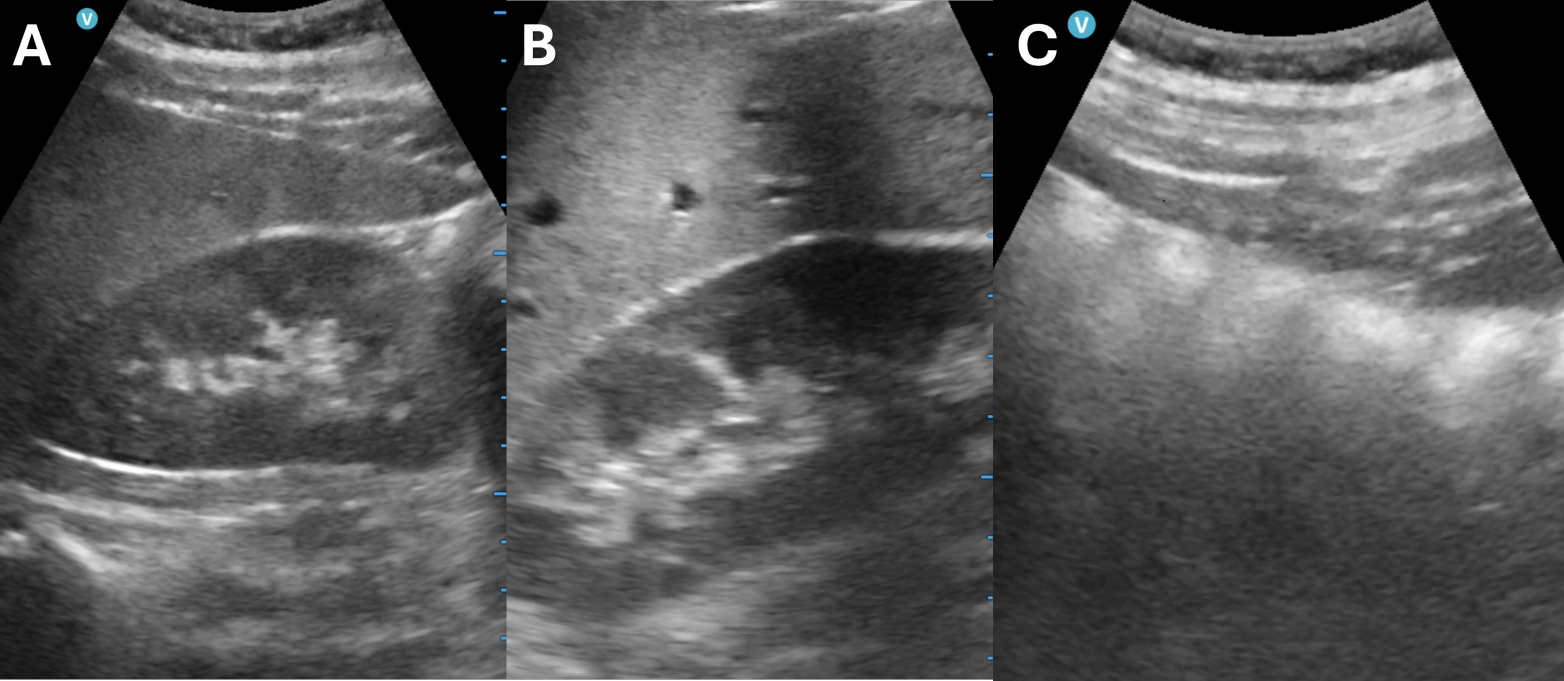
Examples of renal ultrasound images for model training: (A) optimal, (B) suboptimal (artifact and incorrect positioning), and (C) incorrect.

All renal ultrasound images (N=2807) in transverse and longitudinal views were retrospectively retrieved from the local radiology database, anonymized, and used to train the algorithm. All renal ultrasound images were classified into three main categories by a board-certified radiologist with more than 15 years of postfellowship experience. Images were classified as optimal when they were free of artifact and demonstrated appropriate brightness and positioning of the kidney (Figure 1A). Images were classified as suboptimal when they exhibited one or more of the following features: artifact (eg, acoustic shadowing or edge artifact) that obscured visualization of the kidney; incorrect gain (brightness), defined as improper amplification of the ultrasound signal, resulting in either very dark or very bright pixels and a degraded grayscale image; and/or incorrect positioning, in which the image of the kidney was truncated or its contour was unclear (Figure 1B). Images were classified as incorrect when the image showed an incorrect organ or when no kidney was visualized (Figure 1C).

In the training dataset, 562 images were classified as optimal, 1288 as suboptimal, and 957 images as incorrect. Among the 1288 suboptimal images, 200 showed artifact alone, 256 showed incorrect gain alone, 202 showed incorrect position alone, 248 showed artifact and incorrect gain, 106 showed artifact and incorrect positioning, 164 showed incorrect gain and incorrect positioning, and 112 showed all 3 subcategories.

### Two-Stage Cascaded Network

**Figure 2.**
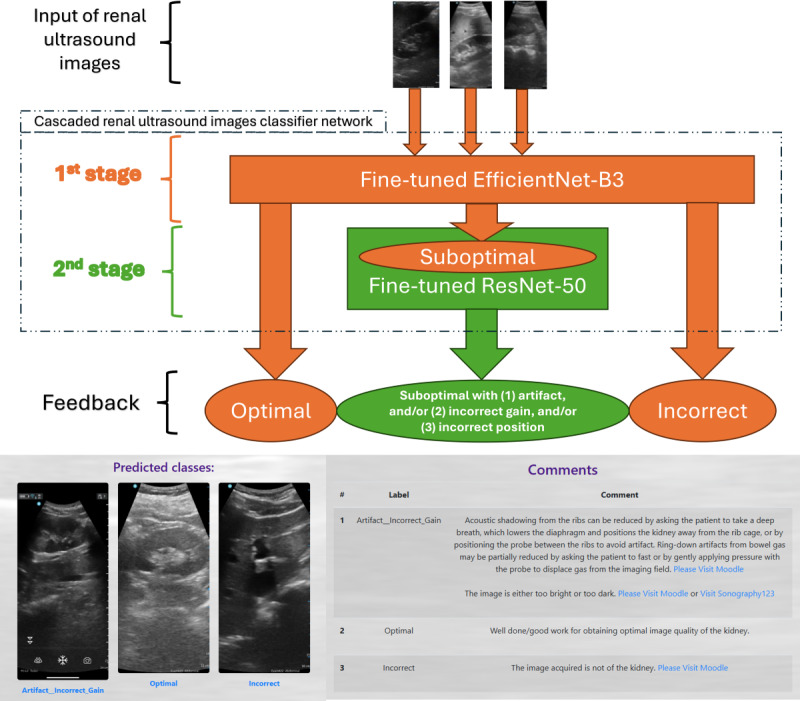
Cascaded renal ultrasound image grading system with automated grading and feedback for students (demonstration video in Multimedia Appendix 1).

All images were preprocessed with pixel resizing (224 × 224 × 1) and *z* score normalization before being used to fine-tune the pretrained CNNs, with an 8:2 split between the training and test sets. The resulting model formed a deep learning–based, fully automated renal ultrasound image grading system (Figure 2). The cascaded classifier network was composed of two stages. In the first stage, a pretrained CNN, EfficientNet-B3 [28], was fine-tuned on all 2807 ultrasound images for the classification task, labeled according to the three previously defined categories. In the second stage, images classified as suboptimal were input into another pretrained CNN, ResNet-50 [26], to further subclassify them into the following subcategories: artifact, incorrect gain, and/or incorrect positioning.

Both EfficientNet-B3 and ResNet-50 backbones pretrained on the ImageNet dataset [29] were used and fine-tuned on the renal ultrasound dataset via transfer learning. Subsequently, the trained model was hosted on a website through Microsoft Azure, the cloud computing platform (Figure 2).

### Study Cohort and Interventions

The deep learning–based feedback model was deployed to a cohort of novice year 5 medical students receiving training in ultrasound imaging during the 2023-2024 academic year. The students received a 1-hour didactic lecture and a 3-hour face-to-face ultrasound training session with an experienced ultrasound instructor. Students were given access to individual ultrasound handheld devices for practice during a 6-week surgical rotation. Students were encouraged to practice ultrasound scanning with their peers and to submit renal ultrasound images to the online platform during the 6-week surgical rotation. Images captured during these practice sessions were uploaded to the online platform, where the model immediately analyzed the submitted images and provided instant feedback and grading. For suboptimal or incorrect images, the platform provided comments that were cross-referenced to the current teaching material on the e-learning platform (Figure 2). Accordingly, students were able to assess image quality and adjust their image scanning technique for subsequent scans. Students had free access to the platform, with no limit on the number of times they could access it or the number of images they could submit, to encourage use during training.

### Mixed Methods Analysis: Qualitative Analysis

Qualitative evaluation was conducted through focus group interviews with selected subgroups within the cohort to gain insight into the successes and challenges of implementing a deep learning–based feedback model in ultrasound training. Two teaching associates were invited to attend the focus group interviews and took notes. These notes were subsequently summarized and circulated among the instructors and teaching associates to ensure crucial points were accurately captured. Thematic analysis was then performed to identify and analyze the pertinent points discussed during the focus group interviews.

### Mixed Methods Analysis: Quantitative Analysis

Students were invited to complete an 11-item questionnaire based on a 5-point Likert scale (Multimedia Appendix 2). All items were selected from validated instruments, including the Objective Structured Assessment of Ultrasound Skills [30], the System Usability Scale [31], and the Client Satisfaction Questionnaire-8 [32]. The questionnaire aimed to evaluate the model’s effectiveness in supporting ultrasound training (items 1‐3), its usability (items 4 and 5), and students’ experiences interacting with the model (items 6‐11). Three experienced medical educators skilled in ultrasound teaching (PYN, MTHC, and EYPL) rated each questionnaire item for relevance to ultrasound learning. All items achieved an Item-Content Validity Index of 1, indicating high relevance to ultrasound education. For reliability testing, 30 medical students were invited to complete the questionnaire twice with a 1-week interval. Cronbach α was 0.965, and the intraclass correlation coefficient was 0.971. Both measures demonstrated high questionnaire reliability.

### Objective Structured Clinical Examination

At the end of the surgical rotation, an objective structured clinical examination (OSCE) was conducted, which included a station assessing renal ultrasound acquisition skills. Students were evaluated at the standardized OSCE station and instructed to perform POCUS on a healthy volunteer to demonstrate normal renal anatomy. The skills assessed included (1) patient preparation, (2) ultrasound probe selection, (3) probe handling, (4) a systematic approach to examination, and (5) the acquisition of an optimal sonographic image. All students were assessed by a surgical specialist experienced in performing POCUS examinations.

Mean OSCE scores from the 2 academic years preceding implementation of the deep learning–based feedback model (2021‐2022 and 2022‐2023) were retrieved and compared with the mean OSCE score of this cohort of students receiving the deep learning–based feedback.

## Results

### Overview

The cohort of year 5 medical students (n=231) receiving ultrasound training was divided into 6 groups and enrolled between October 2023 and September 2024. A total of 786 renal ultrasound images were submitted to the platform for grading after exclusion of duplicate images. The mean number of images contributed per student was 3.40 (SD 3.32). The mean interval between the first submission and the OSCE was 15.6 (SD 6.04) days. Of the 786 images, 269 images were classified as optimal, 349 as suboptimal, and 168 as incorrect.

### Thematic Analysis in Focus Group Interviews

Within the cohort, 2 subgroups were invited for focus group interviews (n=71). Through the interviews conducted in this study, highly motivated students who wanted to be more involved in the project were identified, and these students provided valuable suggestions for model refinement and implementation. The thematic framework identified three major themes: the positive impact of deep learning–based feedback, the challenges of implementation, and suggestions to enhance the feedback model.

#### Positive Impact

Participants generally agreed that the model motivated their learning and helped improved ultrasound skill acquisition. Students were satisfied with the availability of immediate feedback upon submitting images to the platform, which promoted and encouraged self-regulated learning. Representative participant quotations illustrating the positive impact of the model are provided below.

I can know the quality of my images in a short time.

[Participant #16, female]

The system instantly confirmed if my images were qualified.

[Participant #25, male]

Great that it can classify my images immediately.

[Participant #42, male]

It allows me to take more images during practice and upload them later.

[Participant #45, male]

#### Challenges

Two major challenges were identified: curricular design and hardware provision. Students reported infrequent use of the handheld ultrasound device due to limited tutor guidance at the bedside, which reduced the incentive to practice newly acquired skills. In addition, students found the ultrasound curriculum overwhelming in terms of knowledge load and skills mastery. Usability issues with the handheld devices were also reported. Representative participant quotations illustrating these challenges are presented below.

The handheld ultrasound device is different from the one I learned in the lesson.

[Participant #11, female]

I cannot set-up the software of the handheld ultrasound device on my smartphone.

[Participant #13, male]

The battery life of the handheld ultrasound device is not long enough.

[Participant #27, male]

The handheld device needs to be set-up first and it is a bit complicated.

[Participant #52, female]

#### Student Suggestions

Students suggested that integrating real-time feedback would enhance ease of use and potentially increase learning engagement. Representative participant statements are presented below.

Real-time feedback would be more convenient.

[Participant #5, female]

If it provides real-time feedback, then there is no need to take screenshots and upload images.

[Participant #27, male]

### Questionnaire

A response rate of 42.4% was achieved, with 98 of 231 students completing the online questionnaire (Table 1).

**Table 1.** Questionnaire and results (n=98).

Questionnaire item	Respondent scores, n (%)				
	1	2	3	4	5
Q1. The grading system assists me in familiarizing myself with the handheld device and its function.^a^	0 (0)	12 (12)	50 (51)	28 (29)	8 (8)
Q2. The grading system assists me in optimizing image quality.^b^	0 (0)	25 (25)	42 (43)	27 (28)	4 (4)
Q3. The grading system assists me in presenting the renal image according to instruction.^c^	1 (1)	15 (15)	35 (36)	43 (44)	4 (4)
Q4. I would like to use this system frequently.^d^	3 (3)	7 (7)	34 (35)	44 (45)	10 (10)
Q5. The system was easy to use.^d^	1 (1)	2 (2)	21 (21)	53 (54)	21 (21)
Q6. The system was consistent with its grading.^d^	2 (2)	4 (4)	44 (45)	42 (43)	6 (6)
Q7. The system offered useful comments.^d^	1 (1)	9 (9)	36 (37)	44 (45)	8 (8)
Q8. The system met my needs.^d^	1 (1)	6 (6)	37 (38)	46 (47)	8 (8)
Q9. The system helped me learn and improve my ultrasound skills.^d^	1 (1)	6 (6)	26 (27)	56 (57)	9 (9)
Q10. I will recommend this system to other peers.^d^	1 (1)	5 (5)	32 (33)	51 (52)	9 (9)
Q11. I am satisfied with the system.^d^	1 (1)	2 (2)	35 (36)	54 (55)	6 (6)

a Question 1 response options: 1=unable to operate equipment, 2=limited ability to operate equipment, 3=operates with some experience, 4=confident in operating equipment, 5=familiar with operating equipment.

b Question 2 response options: 1=unable to optimize, 2=limited ability to optimize, 3=competent but optimization inconsistently done, 4=confident in optimization with minor inconsistencies, 5=consistent optimization.

c Question 3 response options: 1=unable to achieve, 2=occasionally achieve with difficulty, 3=partially achieve, 4=frequently achieve with some consistency, 5=consistently achieve.

d Questions 4-11 response options: 1=strongly disagree, 2=disagree, 3=neutral, 4=agree, 5=strongly agree.

When evaluating the feedback model for assisting ultrasound skill acquisition (questions 1‐3), 32% (31/98) to 48% (47/98) of the respondents rated the items as 4 or higher, indicating that the model helped build confidence in acquiring new skills, including use of the handheld ultrasound device, image optimization, and image presentation according to instructions. A similar proportion of students (35/98, 36% to 50/98, 51%) rated questionnaire items 1 to 3 as 3, suggesting partial improvement in ultrasound acquisition skills through the feedback from the model (Table 1).

Among respondents, at least 55% (54/98) were satisfied with the usability of the model (scores ≥4 for questions 4 and 5) and more than 49% (48/98) had positive experiences interacting with the model on the cloud platform (scores ≥4 for questions 6‐11). However, 6% (6/98) of students rated the model as inconsistent in its grading (scores 1 or 2 for question 6) and 45% (44/98) were neutral (score 3 for question 6) on this aspect (Table 1).

### OSCE Score Comparison

The mean OSCE score in the current cohort was 9.73 (SD 0.76) out of 10, reflecting high performance in renal ultrasound acquisition skills. In the preceding 2 academic years, when a deep learning–based feedback model was not used, mean OCSE scores were 9.35 (SD 1.03; *P*=.06) out of 10 in the 2021-2022 academic year and 9.45 (SD 0.97; *P*=.15) out of 10 in the 2022-2023 academic year. Although the mean score was higher in the current cohort, the difference was not statistically significant. This finding may reflect the model’s role in sustaining students’ interest in renal ultrasound learning by providing continuous access to the platform and encouraging active engagement during the clinical placement.

## Discussion

### Principal Findings

This study developed and deployed a deep learning–based feedback model to assist novice learners in mastering ultrasound acquisition skills. Artificial intelligence (AI) has already substantially influenced many aspects of society, including medical education [33,34]. Several POCUS studies have explored the use of AI in educational design. Medical students using AI-based tools demonstrated improved performance in acquiring cardiac views on echocardiography [35-38]. Artificial intelligence has also enhanced novice performance in measuring left ventricular ejection fraction, achieving diagnostic accuracy comparable to that of cardiologists [39]. The diagnostic performance of inexperienced medical residents or fellows in the evaluation of thyroid nodules was improved with an AI-based computer-assisted diagnostic system [40]. Integration of AI into 3-dimensional and 4-dimensional ultrasound analysis has enhanced fetal facial profiling, contributing to education in prenatal diagnosis [41]. Collectively, these studies highlight the importance of AI in ultrasound education. The findings of this study are consistent with this evidence, suggesting that a deep learning–based feedback model can serve as an effective adjunct to ultrasound learning by providing automated feedback that supports students’ self-regulated learning.

The positive experiences students reported while interacting with the model were essential in sustaining continuous interest in learning ultrasound skills. According to the 4-phase model of interest development [42], learners progress from triggered situational interest to maintained situational interest and eventually to emerging and well-developed individual interest. The initial face-to-face ultrasound training may have triggered situational interest, whereas the feedback model may have contributed to maintaining that interest, thereby allowing for individual interest to develop.

The deep learning–generated grading was not intended as a final assessment of learning; rather, ambiguous images were encouraged to be reviewed and discussed with instructors during face-to-face sessions. Feedback model analytics (ie, the number of platform accesses and image submissions) indicated that students actively engaged with the model throughout the surgical rotation. These findings suggest that the feedback model promoted self-regulated learning and allowed students to develop ultrasound skills at their own pace [18].

Based on feedback model analytics, students frequently submitted more than 1 renal ultrasound image to the platform for evaluation, which may indicate that these students perceived the model as helpful in supporting their learning. Overall, sustained engagement with the feedback model suggests that it contributed to fostering novice learners’ interest and motivation, and self-regulated their development of ultrasound skills.

Information gathered from the focus group interviews prompted further in-depth discussion among the course instructors and tutors to re-examine the current ultrasound curriculum. Options for streamlining the curriculum into more focused areas are being actively explored. The framework of load reduction [43] may be applied through instructional strategies such as increasing scaffolding and progressively guiding learners toward independent mastery.

To promote student engagement, the benefits of adopting the deep learning–based feedback model and its role in the broader ultrasound curriculum can be communicated to students. Course leaders may also develop reflective tasks or prompts based on principles of self-regulated learning [23] to guide students in reflecting on their learning (ie, self-efficacy, self-monitoring, self-evaluation, adaptive changes) [44]. These reflections can span both face-to-face ultrasound training and interactions with the deep learning–based feedback model during the 6-week surgical rotation. Such integrations may scaffold students’ reflection on their ultrasound learning, help close the feedback loop, and encourage feedforward learning [45]. This refinement to the curriculum structure may enhance self-regulated learning and provide a sustainable, iterative process for students to develop their ultrasound skills through integration of the deep learning–based feedback model.

Furthermore, interview findings indicated a need for enhanced tutor guidance. In addition to scaling-up the recruitment of ultrasound instructors and strengthening local training support, a midrotation tutorial or workshop may provide support to students who may be struggling. This approach may enable them to make more effective use of the feedback model and feel empowered to practice their ultrasound skills more during the rotation. Integrating near-peer feedback during the midrotation tutorial may further support struggling students and help bridge the gaps between intensive face-to-face sessions [46].

As noted from the focus group interviews, full realization of the intervention’s benefits would likely require real-time integration of the model into the hardware device. Real-time integration could address the need for immediate guidance while also providing instructional scaffolding. However, such development would be more resource intensive, requiring real-time image tracking and continuous feedback from the deep learning–based model. With further research, clinical validation, and implementation, this type of AI application could potentially be adopted in POCUS training and possibly incorporated into the OSCE.

There is also room for further improvement such as incorporating highlighted contours or bounding boxes to delineate the kidney and associated artifacts. This could be achieved by integrating additional deep learning–based segmentation or object detection models.

### Limitations

Although the performance of the trained cascaded network was satisfactory for the classification task, there remains room for improvement in its accuracy and efficiency. We are currently prospectively collecting ultrasound data submitted by students to enable continuous model training and refinement, in order to enhance the model’s accuracy and relevance. Second, the response rate to the questionnaire was low, introducing a risk of sampling bias [47]. Despite numerous reminders and encouragement from instructors, students who participated in the questionnaire were likely highly motivated and may have been more proficient in self-regulated learning. Future studies that include a broader range of students would provide a clearer understanding of how the feedback model supports both high- and low-achieving learners in developing ultrasound skills. Third, the lack of a preintervention questionnaire limits the strength of inferences regarding the impact of the deep learning–based model on ultrasound learning and precludes detailed assessment of change. However, given the low postintervention response rate, adding a preintervention questionnaire might have increased the risk of attrition bias.

### Conclusion

A cascaded renal ultrasound image feedback model was successfully developed and deployed, personalizing the learning experience in medical education and providing on-demand feedback. It was well received by students and supported self-regulated learning. The innovation enhanced student engagement and improved ultrasound skill acquisition among novice learners.

## Supplementary material

10.2196/72110 Multimedia Appendix 1 Demonstration video of the cascaded renal ultrasound image grading system.

10.2196/72110 Multimedia Appendix 2 Sample questionnaire.

## Abbreviations

AI: artificial intelligence
CNN: convolutional neural network
OSCE: objective structured clinical examination
POCUS: point-of-care ultrasound
UME: undergraduate medical education

## References

[1] Smallwood N, Dachsel M (2018). Point-of-care ultrasound (POCUS): unnecessary gadgetry or evidence-based medicine?. Clin Med (Northfield).

[2] Archer J, Beck S (2023). Accuracy and clinical use of biliary point-of-care ultrasound: a retrospective cohort study. Emerg Med Australas.

[3] Yoshida T, Yoshida T, Noma H, Nomura T, Suzuki A, Mihara T (2023). Diagnostic accuracy of point-of-care ultrasound for shock: a systematic review and meta-analysis. Crit Care.

[4] Nicholas E, Ly AA, Prince AM, Klawitter PF, Gaskin K, Prince LA (2021). The current status of ultrasound education in United States medical schools. J Ultrasound Med.

[5] Wong CK, Hai J, Chan KYE (2021). Point-of-care ultrasound augments physical examination learning by undergraduate medical students. Postgrad Med J.

[6] Cheung ACK, Ng PY, Lam RPK, Wong GTC (2024). Cross-specialty point-of-care ultrasound education in The University of Hong Kong. Hong Kong Med J.

[7] Russell FM, Zakeri B, Herbert A, Ferre RM, Leiser A, Wallach PM (2022). The state of point-of-care ultrasound training in undergraduate medical education: findings from a national survey. Acad Med.

[8] Breunig M, Hanson A, Huckabee M (2023). Learning curves for point-of-care ultrasound image acquisition for novice learners in a longitudinal curriculum. Ultrasound J.

[9] Moga T, Dancu GM, Cotrau R (2024). Learning curves in abdominal ultrasound in medical students. Med Ultrason.

[10] Alerhand S, Choi A, Ostrovsky I (2020). Integrating basic and clinical sciences using point-of-care renal ultrasound for preclerkship education. MedEdPORTAL.

[11] Burgess A, van Diggele C, Roberts C, Mellis C (2020). Feedback in the clinical setting. BMC Med Educ.

[12] Carless D (2015). Excellence in University Assessment: Learning from Award-Winning Practice.

[13] Tai J, Ajjawi R, Boud D, Dawson P, Panadero E (2018). Developing evaluative judgement: enabling students to make decisions about the quality of work. High Educ.

[14] Bienstock JL, Katz NT, Cox SM (2007). To the point: medical education reviews--providing feedback. Am J Obstet Gynecol.

[15] Reddy ST, Zegarek MH, Fromme HB, Ryan MS, Schumann SA, Harris IB (2015). Barriers and facilitators to effective feedback: a qualitative analysis of data from multispecialty resident focus groups. J Grad Med Educ.

[16] Henderson M, Phillips M, Ryan T (2019). Conditions that enable effective feedback. High Educ Res Dev.

[17] Henderson M, Ryan T, Phillips M (2019). The challenges of feedback in higher education. Assess Eval High Educ.

[18] Fuller R, Goddard VCT, Nadarajah VD (2022). Technology enhanced assessment: Ottawa consensus statement and recommendations. Med Teach.

[19] Cutrer WB, Miller B, Pusic MV (2017). Fostering the development of master adaptive learners: a conceptual model to guide skill acquisition in medical education. Acad Med.

[20] Pickering JD (2015). Anatomy drawing screencasts: enabling flexible learning for medical students. Anat Sci Educ.

[21] Fitzgerald DA, Scott KM, Ryan MS (2022). Blended and e-learning in pediatric education: harnessing lessons learned from the COVID-19 pandemic. Eur J Pediatr.

[22] Challis M (2000). AMEE medical education guide no. 19: personal learning plans. Med Teach.

[23] Zimmerman BJ (2002). Becoming a self-regulated learner: an overview. Theory Pract.

[24] Panadero E (2017). A review of self-regulated learning: six models and four directions for research. Front Psychol.

[25] Krizhevsky A, Sutskever I, Hinton GE (2017). ImageNet classification with deep convolutional neural networks. Commun ACM.

[26] He K, Zhang X, Ren S, Sun J Deep residual learning for image recognition.

[27] Hu J, Shen L, Sun G Squeeze-and-excitation networks.

[28] Tan M, Le QV (2019). EfficientNet: rethinking model scaling for convolutional neural networks. Proc Machine Learning Res.

[29] Deng J, Dong W, Socher R, Li LJ ImageNet: a large-scale hierarchical image database.

[30] Tolsgaard MG, Todsen T, Sorensen JL (2013). International multispecialty consensus on how to evaluate ultrasound competence: a Delphi consensus survey. PLoS One.

[31] Brooke J. SUS, Jordan PW, Thomas B, McClelland IL, Weerdmeester B (1996). Usability Evaluation in Industry.

[32] Larsen DL, Attkisson CC, Hargreaves WA, Nguyen TD (1979). Assessment of client/patient satisfaction: development of a general scale. Eval Program Plann.

[33] Tolsgaard MG, Pusic MV, Sebok-Syer SS (2023). The fundamentals of artificial intelligence in medical education research: AMEE Guide No. 156. Med Teach.

[34] Gordon M, Daniel M, Ajiboye A (2024). A scoping review of artificial intelligence in medical education: BEME guide no. 84. Med Teach.

[35] Gohar E, Herling A, Mazuz M (2023). Artificial intelligence (AI) versus POCUS expert: a validation study of three automatic AI-based, real-time, hemodynamic echocardiographic assessment tools. J Clin Med.

[36] Aronovitz N, Hazan I, Jedwab R (2024). The effect of real-time EF automatic tool on cardiac ultrasound performance among medical students. PLoS One.

[37] Baum E, Tandel MD, Ren C (2023). Acquisition of cardiac point-of-care ultrasound images with deep learning: a randomized trial for educational outcomes with novices. Chest Pulmonary.

[38] Soliman-Aboumarie H, Geers J, Lowcock D (2025). Artificial intelligence-assisted focused cardiac ultrasound training: a survey among undergraduate medical students. Ultrasound.

[39] Dadon Z, Orlev A, Butnaru A (2023). Empowering medical students: harnessing artificial intelligence for precision point-of-care echocardiography assessment of left ventricular ejection fraction. Int J Clin Pract.

[40] Lee SE, Kim HJ, Jung HK (2024). Improving the diagnostic performance of inexperienced readers for thyroid nodules through digital self-learning and artificial intelligence assistance. Front Endocrinol (Lausanne).

[41] Bachnas MA, Andonotopo W, Dewantiningrum J, Adi Pramono MB, Stanojevic M, Kurjak A (2024). The utilization of artificial intelligence in enhancing 3D/4D ultrasound analysis of fetal facial profiles. J Perinat Med.

[42] Hidi S, Renninger KA (2006). The four-phase model of interest development. Educ Psychol.

[43] Martin AJ, Evans P (2018). Load reduction instruction: exploring a framework that assesses explicit instruction through to independent learning. Teach Teach Educ.

[44] Leggett H, Sandars J, Roberts T (2019). Twelve tips on how to provide self-regulated learning (SRL) enhanced feedback on clinical performance. Med Teach.

[45] Fuentes-Cimma J, Sluijsmans D, Riquelme A (2024). Designing feedback processes in the workplace-based learning of undergraduate health professions education: a scoping review. BMC Med Educ.

[46] Sader J, Cerutti B, Meynard L (2022). The pedagogical value of near-peer feedback in online OSCEs. BMC Med Educ.

[47] Spooner M, Pawlikowska T (2023). Feedback literacy as a model to explore how learners respond to feedback. Br J Hosp Med.

