# Supplementary material for: Development of a Deep Learning–Based Feedback Model to Assist Medical Students Learning Renal Ultrasound Acquisition: Mixed Methods Study
Source: JMIR Med Educ. 2026 Mar 9;12:e72110. doi: 10.2196/72110 (PMC12978925; doi:10.2196/72110)
Supplement: Multimedia Appendix 2 [file mededu-v12-e72110-s002.docx]

Multimedia Appendix 2: Sample questionnaire

Questionnaire for Renal Ultrasound Image Grading System

Q1) The grading system assists me in familiarising myself with the handheld device and its function

1. Unable to operate equipment
2. Limited ability to operate equipment
3. Operates with some experience
4. Confident in operating equipment
5. Familiar with operating

Q2) The grading system assists me in optimising image quality

1. Unable to optimize
2. Limited ability to optimize
3. Competent but inconsistently done
4. Confident in optimization with minor inconsistencies
5. Consistent optimization

Q3) The grading system assists me in presenting the renal image according to instruction

1. Unable to achieve
2. Occasionally achieve with difficulty
3. Partially achieve
4. Frequently achieve with some consistency
5. Consistently achieve

Q4) I would like to use this system frequently

1. Strongly disagree
2. Disagree
3. Neutral
4. Agree
5. Strongly agree

Q5) The system was easy to use

1. Strongly disagree
2. Disagree
3. Neutral
4. Agree
5. Strongly agree

Q6) The system was consistent with its grading

1. Strongly disagree
2. Disagree
3. Neutral
4. Agree
5. Strongly agree

Q7) The system offered useful comments

1. Strongly disagree
2. Disagree
3. Neutral
4. Agree
5. Strongly agree

Q8) The system met my need

1. Strongly disagree
2. Disagree
3. Neutral
4. Agree
5. Strongly agree

Q9) The system helped me learn and improve ultrasound skills

1. Strongly disagree
2. Disagree
3. Neutral
4. Agree
5. Strongly agree

Q10) I will recommend this system to other peers

1. Strongly disagree
2. Disagree
3. Neutral
4. Agree
5. Strongly agree

Q11) I am satisfied with the system

1. Strongly disagree
2. Disagree
3. Neutral
4. Agree
5. Strongly agree
